# Abnormal Expression of E-Cadherin in Gastric Adenocarcinoma, and Its Correlation With Tumor Histopathology and Helicobacter Pylori Infection

**DOI:** 10.5812/ircmj.4032

**Published:** 2013-03-05

**Authors:** Robab Anbiaee, Khosrow Mojir Sheibani, Peyman Torbati, Hanieh Jaam

**Affiliations:** 1 Oncology Department, Imam Hossein Hospital, Shahid Beheshti University of Medical Sciences, Tehran, IR Iran; 2 Radiotherapy and Oncology Department, Imam Hossein Hospital, Shahid Beheshti University of Medical Sciences, Tehran, IR Iran; 3 Department of Pathology, Labafi Nejad Hospital, Shahid Beheshti University of Medical Sciences, Tehran, IR Iran; 4 Department of Pathology, Shohada-e-Tajrish Hospital, Shahid Beheshti University of Medical Sciences, Tehran, IR Iran

**Keywords:** Stomach Neoplasms, Cadherin, Helicobacter Pylori

## Abstract

**Background:**

Gastric cancer is one of the leading cancers in the world especially in Iran. There are many genomic and molecular factors that cause gastric cancer to occur, and also there are many markers that associate with tumor invasiveness. E-cadherin is a tumor suppressor gene which produces E-cadherin transmembrane protein, a molecule which plays an important role in adhesion and differentiation of epithelial cells.

**Objectives:**

In this study, we evaluated the prevalence of abnormal E-cadherin expression in Iranian patients with gastric adenocarcinoma, and tried to find its correlation with H. pylori infection and tumor histopathology.

**Materials and Methods:**

A historical cohort survey was performed on tissue samples obtained from 95 total or partial gastrectomy with gastric adenocarcinoma. The immunohistochemistry and Giemsa staining were used to assess E-cadherin expression, and H- pylori infection respectively. The association between abnormal E-cadherin expression and tumor histopathology characteristics include depth of tumor invasion, tumor differentiation, tumor phenotype, tumor type, tumor size, neurovascular invasion of tumor, and regional lymph node involvement and H- pylori infection and patient's age and sex, were evaluated in all patients.

**Results:**

Abnormal E-cadherin expression was noted in 38% of patients, and 59% of patients were infected with H-pylori. A significant correlation was seen between abnormal E-cadherin expression, and tumor grade and regional lymph node involvement. We could not find any significant association between abnormal E-cadherin expression and H- pylori infection, patient's age and sex, tumor phenotype, tumor type, depth of tumor invasion, tumor size, and neurovascular invasion of tumor.

**Conclusions:**

Abnormal E-cadherin expression is a common phenomenon in gastric adenocarcinoma. The study showed a significant correlation between abnormal E-cadherin expression and tumor grade and regional lymph node involvement; so, abnormal E-cadherin expression may be used as a predictive factor for tumor invasiveness in gastric adenocarcinoma.

## 1. Background

Gastric cancer is one of the most common malignancies in the world, and it is especially prevalent in Iran (1). E-cadherin gene is a tumor suppressing gene, expressing E-cadherin transmembrane glycoprotein, which plays a significant role in adhesion and differentiation of epithelial cells (1). Any mutation in this gene can result in a grate defect in cell adhesion, and considered as a starting point for cancer occurrence. It is postulated that the environmental factors may cause cancer by impeding the action of E-cadherin gene (1-3). Also, it has been observed that gastric cancers with mutated E-cadherin gene are more invasive than the other types of gastric cancer without this mutation (4-7). It might be related to the defect in cell adhesion, an important factor for metastasis (1). The etiological factors in gastric cancer are generally categorized into three main groups: a) acquired factors; b) genetic factors; and c) cancer promoting lesions (1, 2). Genetic factors are involved in < 10% of gastric cancers, while most of the malignancies result from acquired factors. Gastric infection with H. pylori is one of the most important acquired factors. WHO has classified it as class I group carcinogens. It is associated with greater risk of gastric adenocarcinoma, and gastric lymphoma (6, 7), but it is still unclear how H. pylori results in development of gastric cancer (1, 7). This study was performed to investigate the association between alteration in E-cadherin expression and histopathological characteristics of gastric adenocarcinoma in patients who undergone gastrectomy because of gastric adenocarcinoma. Also, we evaluated the association between H. pylori infection as an acquired risk factor for gastric cancer, and alteration in E-cadherin expression as the probable mechanism for development of cancer.

## 2. Objectives

In this study, we evaluated the prevalence of abnormal E-cadherin expression in Iranian patients with gastric adenocarcinoma, and tried to find its correlation with H. pylori infection and tumor histopathology.

## 3. Materials and Methods 

This historical cohort survey was performed on 95 gastric tumors obtained from patients with documented gastric adenocarcinoma who underwent total or partial gastrectomy, and regional lymphadenectomy. No patient had received any adjuvant therapy (neither chemotherapy nor radiotherapy) before surgery. Because we only worked with gastrectomy samples without knowing the name of patients, there was no need to take informed consent. The study was approved by the ethics committee of Shahid Beheshti University of Medical Sciences. Gastric tumors other than adenocarcinoma (e.g. gastric lymphoma), and also tumors that their blocks or slides were not suitable for IHC staining or Giemsa staining were not included in the study. Information about age and sex were obtained from patients records at pathology department. Histopathological factors of the tumor including depth of tumor invasion, size, grade and phenotype of tumor, vascular, lymphatic or neurological invasion and regional lymph nodes involvement were extracted from pathology reports. IHC was used for assessing the expression of E-Cadherin by this method: Formalin-fixed, paraffin-embedded blocks of tumors were sectioned at a nominal (3, 5) micrometer. Heat- induced antigen retrieval using microwave method was applied for all staining. In details, the blocks were deparaffinized and processed as follows: 1) the samples were placed in oven at 37◦C for 48 hour, 2) the samples were rinsed in 100% xylol, ethanol 100%, 85%, 75%, and distilled water, 3) rinsed in PBS solution, 4) exposed to H2O2 10%, and methanol at a ratio of 1:9 for 30 min, 5) rinsed in PBS, 6) placed in citrate buffered solution (pH = 6) for 14 min at a microwave with power 800, 7) rinsed in PBS, 8) blocking serum was added to slides for 30 min, and then dried, 9) addition of E-Cadherin antibody for 30 min at room temperature, 10) rinsed in PBS, 11) addition of Envision + visualization system (Dako) , 12) addition of DAB for 10 min, 13) rinsed in PBS, and dehydrated in alcohol, and finally counterstained with hematoxylin. The slides were evaluated under light microscopy. Appropriate positive and negative controls omitting the primary antibodies were included with each slide run. The H. pylori infection status also was determined by Giemsa staining on nontumoral segments of the stomach. The frequency of abnormal E-cadherin expression and H. pylori infection were determined, and analyzed statistically. To describe data, we used mean ± standard deviation, Median (Range), frequency and percent, odds ratio, and 95% confidence interval (95% CI). To evaluate the differences between two groups when considering the cluster effect of hospitals we used generalized estimating equation (GEE). SPSS software version 17 was used for statistical analysis and the level of significance was 0.03.

## 4. Results

It was a historical cohort study on gastric adenocarcinoma tumors. Totally 95 gastric cancer specimens from patients with gastric adenocarcinoma diagnosis were studied with the mean ± SD age of 60.2 ± 13.3 (median: 62, range: 19 to 85) years. Among them 68 (71.6%) patients were male and 27 (28.4%) were female. Abnormal E-cadherin expression was noted in 38% of patients. Considering the degree of tumor differentiation, patients were divided into 2 groups: low grade (G_1_, G_2_), and high grade (G_3_, G_4_). In each group, expression of E-cadherin was investigated. Abnormal E-cadherin expression among high grade tumors was more than low grade ones. (63% vs. 36%; P = 0.016) (Table 1). Abnormal E-cadherin expression was correlated with high grade tumors. Regarding the number of excised regional lymph nodes, patients were divided into three groups: 5 or less than 5, 6-9, and 10 or more than 10 excised regional lymph nodes. In each group, expression of E-cadherin was investigated in patients with and without lymph node involvement. In the group of 6-9 excised lymph nodes, abnormal E-cadherin expression among patients with positive findings for lymph node was much more than those with negative lymph node results(100% vs. 0%; P = 0.01). So in this group, abnormal E-cadherin expression was correlated with lymph node involvement (Table 2). Regarding the depth of tumor invasion, patients were divided into T_1_, T_2_, T_3_, and T_4_. The T_1_ and T_2_ were considered as early T, and T_3_ and T_4_ as advanced T. E-cadherin expression was evaluated in both groups. Any association between depth of tumor invasion and abnormal E-cadherin expression was evaluated, and was not statistically significant (Table 1). Based on the tumor phenotype, patients were divided into two groups: intestinal and diffused types. E-cadherin expression was evaluated in both groups, and the result was not statistically significant; although in the diffuse type tumors, abnormal E-cadherin expression was more than intestinal type (69% v 31%). Similarly, the vascular invasion, neural invasion, sex, age (< 55 or ≥ 55), tumor size (< 5 cm, 5-10 cm, > 5 cm), and pathological type of the tumor (adenocarcinoma or signet ring carcinoma) were not correlated with abnormal E-cadherin expression. In summary, abnormal E-cadherin expression showed a significant correlation with high grade tumors and lymph node involvement (Table 1). Among 94 patients who Giemsa staining for H-pillory were performed 59 patients were affected. Abnormal E-cadherin expression among the patients with positive and negative findings for H-pillory was observed in 17(51.5%), and 16 (48.5%) of patients, respectively. The association between Abnormal E-cadherin expression and H-pillory infection was not statistically significant (Table 1).

**Table 1.** Association Between Abnormal E-Cadherin Expression, and Tumor Histopathologic Characteristics, and H-Pylori Infection

Parameter	Total	E-cadherin		P value
		Normal, No. (%)	Abnormal, No. (%)	
**Early T, No. (%)**	43 (45.3)	28 (45.2)	15 (45.5)	0.978
**Advanced**	52 (54.7)	34 (54.8)	18 (54.5)	
**Grade, No. (%)**				0.016
I + II (low)	50 (53.2)	38 (62.3)	12 (36.4)	
III + IV (High)	44 (46.8)	23 (37.7)	21 (63.6)	
**Phenotype, No. (%)**				
Intestinal	39 (44.8)	30 (51.7)	9 (31.0)	0.067
Diffuse	48 (55.2)	28 (48.3)	20 (69.0)	
**VI^a^, No. (%)**				0.212
No	44 (47.8)	32 (52.5)	12 (38.7)	
Yes	48 (52.2)	29 (47.5)	19 (61.3)	
**Sex, No. (%)**				
Female	27 (28.4)	20 (32.3)	7 (21.2)	0.256
Male	68 (71.6)	42 (67.7)	26 (78.8)	
**T Type, No. (%)**				
Signet ring	20 (21.1)	10 (16.1)	10 (30.3)	0.107
Adenocarcinoma	75 (78.9)	52 (83.9)	23 (69.7)	
**NI^a^, No. (%)**				0.569
No	36 (47.4)	23 (50.0)	13 (43.3)	
Yes	40 (52.6)	23 (50.0)	17 (56.7)	
**Age**				
Mean± SD	60.2 ± 13.2	60.2 ± 12.7	60.4 ±14.6	0.947
< 55, No. (%)	34 (35.8)	23 (37.1)	11 (33.3)	
> 55, No. (%)	61 (64.2)	39 (62.9)	22 (66.7)	
**Size**				
Mean	6.4 ± 3.1	6.4 ± 3.1	6.5 ± 3.2	0.799
≤ 5.0	46 (48.9)	28 (45.9)	18 (54.5)	0.619
5.1 - 10.0, No. (%)	36 (38.3)	26 (42.6)	10 (30.3)	
< 10, No. (%)	12 (12.8)	7 (11.5)	5 (15.2)	
**H-Pylori, No. (%)**				0.97
Negative	59 (62.28)	19 (31.1)	16 (48.5)	
Positive	35 (37.2)	42 (68.9)	17 (51.5)	

^a^Abbreviations: NI, neural invasion; VI, vascular invasion

**Table 2.** Association Between Abnormal E-Cadherin Expression, and Regional Lymph Node Involvement

Total Lymph Node	Total Patients	E-cadherin		P value
		Normal, No. (%)	Abnormal, No. (%)	
**≤ 5**				0.8
Involved	8	4 (50)	4 (50)	
Not Involved	8	4 (50)	4 (50)	
**6-9**				0.01
Involved	26	17 (73.9)	9 (100)	
Not Involved	6	6 (26.1)	0 (0.0)	
**< 10**				0.33
Involved	34	24 (88)	10 (76.9)	
Not Involved	6	3 (11.1)	3 (23.1)	

## 5. Discussion

E-cadherin is a tumor suppressor gene which is located on chromosome 16 (q 22.1), and produces E-cadherin transmembrane protein. E-cadherin is a calcium-mediated membrane molecule which plays an important role in adhesion and differentiation of gastric epithelial cells, which is a very important protective mechanism against neoplasm formation. E-cadherin gene mutation has been reported in several epithelial cancers with different frequencies (1, 2). In our study, 38% of cases of gastric adenocarcinoma showed abnormal E-cadherin expression in IHC. This frequency among gastric cancer was reported 46% in the study of Dr. Yong-Ning Zhou et al. in china, 3, 58% in the study by Dr. Chan et al. in Hong Kong 5, and 82% in the study of Dr. PabloGuzman et al.(8) These differences may be related to the method of mutation assessment (E-cadherin expression by IHC or E-cadherin gene methylation). In our study absence of membranous and cytoplasmic staining was considered as abnormal E-cadherin expression. Among 95 gastric cancer samples in our study, 94 were assessed for H. pylori infection, and 59% were infected with this microorganism that was not correlated with abnormal E-cadherin expression. Although the association between gastric cancer and this infection has been proved in several studies since several years ago, (1, 2, 9, 10), but the mechanism of causing gastric cancer by H. pylori is still unclear. Maybe chronic infection with H. pylori can promote carcinogenicity by causing mutation in tumor suppressor genes, and perhaps this is the first step in carcinogenicity caused by this microorganism (9, 11). In the study by Chan et al. in Hong Kong, it was proposed that H. pylori might cause E-cadherin mutation, and this mutation can be one of the initial changes in gastric cancer occurrence. Also it was discussed that complete eradication of H-Pylori infection in a metaplastic stomach can stop the changing process in this tumor suppressor gene, and the ensuing cancer11 but they could not find any association between E-cadherin mutation and gastric cancer. This finding in our study and similar studies like Dr. Chan’s study may be related to this fact that these are retrospective studies, and the prior status of H-Pylori infection was not known in all patients. Although we investigate the H-Pylori status on nontumoral area of stomach, but we know that the H-Pylori infection subsides as the cancer progress. Although we could not find the association between H-Pylori infection and gastric cancer, but we should keep in mind that all patients with H. pylori infection must be screened for any gastric metaplasia or gastric cancer, and eradication of H. pylori infection must be confirmed in them. Although there are different results about the association between E-cadherin mutation, and histopathology and tumor invasiveness, but most studies indicate that this mutation is associated with more aggressive tumors such as lobular carcinoma of breast (1). In our study a significant correlation was seen between abnormal E-cadherin expression and tumor grade and regional lymph node involvement, the two parameters which show the invasiveness of tumor. This is an important issue because E-cadherin mutation can be used as a significant prognostic factor, and its detection can be useful in revealing causes of mutation and decreasing the rate of invasive cancers by preventing them. We could not find any association between abnormal E-cadherin expression, and depth of tumor invasion, tumor size, phenotype (diffused or interstitial), and type (adenocarcinoma or signet ring), vascular and lymphatic invasion, neuronal invasion and patient’s age and sex. In the study of Dr. Chan E-cadherin methylation was correlated with depth of tumor invasion and nodal metastasis.5Also in the study of Dr. Jawhari et al. E-cadherin mutation was associated with diffuse type cancer, and it was not correlated with tumor grade or stage (12). In conclusion our study revealed that abnormal E-cadherin expression is associated with more aggressive gastric tumors, and can be used as a negative prognostic factor. Finally we recommend evaluating the association between E-cadherin mutation and overall survival of patients to achieve more definitive results. Also we recommend evaluating the association between H. pylori infection and E-cadherin mutation by genome studying, and other environmental factors that may affect this mutation, so that the rate of invasive gastric cancers may be decreased by preventing them.
